# Correlation of inflammatory and nutritional indexes in Isfahan hemodialysis patients

**Published:** 2010

**Authors:** Omolbanin Kafeshani, Mohammad Hasan Entezari, Mahmood Yahai, Seyed Mohsen Hoseini, Shiva Seiraphian

**Affiliations:** a Nutritionist, Food Security and Nutrition Research Center, Department Of Nutrition, School of Health, Isfahan University Of Medical Sciences, Isfahan, Iran; b Assistant Professor, Food Security and Nutrition Research Center, Department of Nutrition, School of Health, Isfahan University Of Medical Sciences, Isfahan, Iran; c Chemist, Department Of Nutrition, Isfahan University Of Medical Sciences, Isfahan, Iran; d Assistant Professor, Department Of Epidemiology and Biostatistics, School of Health, Isfahan University Of Medical Sciences, Isfahan,, Iran; e Assistant Professor of Nephrology, Department Of Internal Medicine, School of Medicine, Isfahan University Of Medical Sciences, Isfahan, Iran

In recent years, more attention has been focused on inflammatory processes as the possible cause of accelerated atherosclerosis, as well as PEM and concurrent wasting syndrome, which lead to a poor outcome in those with underlying kidney disease.^1^ Malnutrition inflammation syndrome is one of the risk factors of cardiovascular diseases among HD patients, and on the other hand cardio vascular diseases are responsible for 50% of mortality rate among them.^2^ Several investigators suggested that PEM is a consequence of chronic inflammatory and others suggested the role of inflammation as a primary cause of PEM.

This study carried out on 35 HD patients (58.8% male and 48.1% female) referring to dialysis ward of Al-Zahra hospital. These patients were under dialysis for at least 6 months and were dialyzed at least twice a week. At the first, anthropometric characteristics including height and weight were measured and blood sample was taken from patients and sent to the laboratory for clinical tests including cholesterol, triglyceride, albumin, BUN, HG, Hct, and CRP. Demographic information was gathered by questionnaire and the amount of intake nutrients (including protein, fat, carbohydrate, vitamins and minerals) of individuals was investigated by using Food Frequency Questionnaire (FFQ) including 192 food items.

In this study CRP as the inflammation index and albumin and nutrient intake as the nutritional indexes were measured. Of all, 25.9% had albumin less than 3.5 g/dl who were suffering from malnutrition or were exposed to it, and 27.5% had CRP more than 1 mg/dL which is indicative of the existence of inflammation in these people. While there was no meaningful relation between CRP and albumin, (p = 0.38) and between CRP and intake nutrients including protein, fat, carbohydrate, vitamins and minerals and received energy, except for vitamin B1 and selenium, (p = 0.000), therefore, probably because of being short-term, many of the participants in the study haven’t been involved in the inflammation-malnutrition cycle yet.
